# Circad: a comprehensive manually curated resource of circular RNA associated with diseases

**DOI:** 10.1093/database/baaa019

**Published:** 2020-03-27

**Authors:** Mercy Rophina, Disha Sharma, Mukta Poojary, Vinod Scaria

**Affiliations:** 1 Genome Informatics Department, CSIR-Institute of Genomics and Integrative Biology, Mathura Road, District-South Delhi, New Delhi-110025, India; 2 Academy of Scientific and Innovative Research (AcSIR), AcSIR headquarters, CSIR-HRDC campus, Postal Staff College Area, Sector 19, Kamla Nehru Nagar, Ghaziabad, Uttar Pradesh -201002, India

## Abstract

Circular RNAs (circRNAs) are unique transcript isoforms characterized by back splicing of exon ends to form a covalently closed loop or circular conformation. These transcript isoforms are now known to be expressed in a variety of organisms across the kingdoms of life. Recent studies have shown the role of circRNAs in a number of diseases and increasing evidence points to their potential application as biomarkers in these diseases. We have created a comprehensive manually curated database of circular RNAs associated with diseases. This database is available at URL http://clingen.igib.res.in/circad/. The Database lists more than 1300 circRNAs associated with 150 diseases and mapping to 113 International Statistical Classification of Diseases (ICD) codes with evidence of association linked to published literature. The database is unique in many ways. Firstly, it provides ready-to-use primers to work with, in order to use circRNAs as biomarkers or to perform functional studies. It additionally lists the assay and PCR primer details including experimentally validated ones as a ready reference to researchers along with fold change and statistical significance. It also provides standard disease nomenclature as per the ICD codes. To the best of our knowledge, circad is the most comprehensive and updated database of disease associated circular RNAs.

**Availability:** http://clingen.igib.res.in/circad/

## Introduction

Circular RNAs are unique transcript isoforms characterized by a circular conformation and produced by the back-splicing of 5′ donor end and 3′ acceptor ends of pre-mRNA transcripts (1). While the existence of circular isoforms has been previously established for viruses (2), recent evidence suggests they are ubiquitous (3). Circular RNAs have now been reported in a number of organisms including Humans (3), mouse (3), flies (4) and many plants (5). While earlier considered as splicing artefacts (6), recent reports suggest that they are produced through a coordinated biological process and regulated by key genes (7). The mechanism of action has been putatively reported via miRNAs, mRNAs, and RNA-binding proteins (RBPs) (8). For example, CDR1as circRNA has been shown to interact with miR-7 with further effect on cerebral development (3). The role of circular RNA candidates in key biological processes has also been now explored in detail, as in the case of circ-Sry, shown to have a role in sex differentiation (9). Notwithstanding emerging evidence on the functional roles of candidate circular RNAs, the functional annotation of majority of the candidate circular RNAs is still unexplored or needs detailed experimental study to understand their functions.

A number of studies in recent years have analysed the expression patterns of circular RNAs in disease states, and many suggest its potential utility as a biomarker. The closed-loop conformation also provides them an additional advantage as a biomarker that they are resistant to exonuclease digestion (1). These include the studies on a variety of diseases including brain disorders (10), for example, Alzheimer’s disease (11); Parkinson disease (12); different cancers including gastric cancer (12–15), lung cancer (16) and colorectal cancer (16, 17); esophageal carcinoma (18); and adenocarcinoma (19), just to name a few.

The emerging corpus of information, while available as disparate pieces of evidence, would significantly benefit from a unified resource systematically cataloguing disease associations of circular RNAs. This is more significant, given the fact that there are a number of distinct approaches that have been employed to validate and characterize candidate circRNAs including microarray, quantitative RT-PCR and RNA *in situ* hybridization and distinct nomenclature for diseases and traits, which preclude a systematic survey and analysis of existing corpus of evidence on disease associations of circular RNAs.

In the present report, we describe a comprehensive and standardised resource of circular RNAs and disease associations manually curated from literature and following the International Statistical Classification of Diseases (ICD) codes for standardised nomenclature of diseases.

## Results

The manual curation process identified a total of 1388 circRNA transcripts originating from 720 genes and associations with 150 disease conditions mapping to a total of 113 unique disease codes as per ICD. The user interface available at http://clingen.igib.res.in/circad/ allows users to search the collection in a user-friendly manner. The web interface allows the user to query the database in different formats as detailed in the example searches, detailed on the homepage. The user can query the database using names of circRNAs, circRNA aliases, Gene Names, Locus, Organism, Disease, ICD-10 and Pubmed identifiers. A list of circRNAs, matching the search criteria, along with the gene, organism and associated diseases, will be displayed. Detailed information about the circRNA can be obtained by clicking on the name of the circRNA.

The detailed information about each circRNA provided by the resource includes the aliases as well as linkouts to relevant databases. For candidates which have been experimentally validated, the PCR primer sequences are provided, and if not, a list of predicted primers is also provided to aid the researchers. Appropriate linkages are also provided to literature evidence supporting the disease association. A comprehensive user manual is available with details on formatting specific queries and explaining the features, navigation and options available.

## Discussion

A number of complementary resources list disease and trait associations of circular RNAs. The major resources include Circ2Traits (20), circ2disease (21), circRNA disease (22) and CircR2Disease (23). Circ2trait encompasses computational predictions of circular RNAs and disease associations, by virtue of interactions modulated through miRNAs (20). circRNA disease lists 330 disease-associated circular RNAs (22) with 48 diseases, circ2diseases covers only 237 circular RNAs with 217 diseases while circR2Disease lists a total of 661 disease-associated circular RNAs associated with 100 diseases, respectively (23). None of the databases mentions comparison group, and it is important because the higher the sample size, the more the significant biomarker. Also, the abovementioned databases do not mention the fold-change difference and *P* value significance for the upregulated/downregulated expression. Circad not only reports the number of samples and comparison group but also shows the fold change and *P* value. The database has also suggested primers as well as primers used by the respective studies for analysis which is not the case in other databases. Circad lists additional information, including method of validation, status of experimental validation, primer information and above all, a standardised nomenclature using ICD10 codes, thereby enabling standardised interpretation of associations. Circad is updated till December 2019.

## Conclusion

The evidence on circular RNAs and disease associations are continuously emerging, and a process of constant updation forms the key to be relevant and comprehensive. Circad is updated on a regular basis to keep abreast and updated of the relevant evidence as they emerge. In the future, we hope to integrate the database closely with information on expression levels of the circular RNAs in different disease states, as well as information on experimentally validated molecular interactions of circular RNAs with other biomolecules in the cell. This would enable mechanistic understanding of the circular RNA functions in diseases. In short, we foresee and envisage circad as a one-stop and comprehensive resource for circular RNAs associated with diseases and a ready reference in the field of circular RNA biomarkers.

## Materials and methods

### Manual curation of circular RNA-disease associations

Literature databases and resources including PubMed and Google scholar were used to query and retrieve relevant annotations using a combination of keywords. PubMed was queried using standard Query (((circRNA OR circular RNA)) AND “Diseases Category”[Mesh] to retrieve all relevant publications indexed on circular RNAs and diseases. The publications were then systematically checked for information including disease for which the role of circRNA had been reported, along with its method of experimental validation (microarray, qRT-PCR or any other method) and primer information, wherever available. The information was systematically collected in a pre-formatted template. We additionally retrieved information on the fold changes observed among comparison groups, apart from the statistical significance of the observed change. Towards providing a standardised nomenclature for Diseases/Traits, we used the latest ICD-10 nomenclature, which is the 10th revision of the International Statistical Classification of Disease and related health problems by World Health Organization (WHO). Additional information on aliases and links to relevant databases for candidate circRNAs were added through manual comparison.

### Database and web interface

The information from the systematic curation was ported to respective database tables in MySQL. A user-friendly web interface which enables query of the database was built using LAMP stack architecture and hosted on the Apache HTTP server. The user-interface was coded in Perl-CGI and HTML, CSS.

## Author’s contributions

M.W. and D.S. curated the data for database. M.P. designed the database. D.S. designed the primers and wrote the manuscript. V.S. conceived and designed the project. All authors approved the final manuscript.
